# Designing Improved Safer Gambling Messages for Race and Sports Betting: What can be Learned from Other Gambling Formats and the Broader Public Health Literature?

**DOI:** 10.1007/s10899-023-10203-4

**Published:** 2023-03-24

**Authors:** Philip W. S. Newall, Matthew Rockloff, Nerilee Hing, Hannah Thorne, Alex M. T. Russell, Matthew Browne, Tess Armstrong

**Affiliations:** 1 School of Psychological Science, University of Bristol, 12a Priory Road, Bristol, BS8 1TU UK; 2 Experimental Gambling Research Laboratory, School of Health, Medical and Applied Sciences, CQUniversity, 400 Kent St, Sydney, NSW 2000 Australia; 3 Experimental Gambling Research Laboratory, School of Human, Medical, and Applied Sciences, CQUniversity, University Drive, Bundaberg, QLD 4670 Australia; 4 Experimental Gambling Research Laboratory, School of Human, Medical, and Applied Sciences, CQUniversity, 44 Greenhill Rd, Wayville, SA 5034 Australia

**Keywords:** Warning labels, Electronic gambling machines, Gambling products, Gambling-related harm, Wagering, Race betting

## Abstract

Safer gambling messages are one potential input to a public health approach toward reducing gambling-related harm, and yet there is no strong evidence supporting current messages such as “gamble responsibly” or “keep the fun in the game”. Furthermore, sports betting is increasing in popularity in multiple jurisdictions, such as Australia and the US, increasing the need to design effective messaging campaigns for race and sports betting. Compared to other gambling formats, such as electronic gambling machines, the level of potential skill involved in race and sports betting may raise unique issues regarding the design of effective messages. This review first highlights research from the related public health domains of alcohol and tobacco. Then, five potential areas for further messaging-based research in race and sports betting are discussed: teaching safer gambling practices, correcting gambling misperceptions, boosting conscious decision making, norm-based messages, and emotional messages. A broad approach to message design is encouraged, given the potential for individual differences in message receptivity, and for frequently-repeated messages to be ignored or cause negative psychological reactance.

Gambling is increasingly being seen by many researchers and some policymakers as a public health issue, which means adopting a range of strategies to reduce gambling-related harm in the population (Bowden-Jones et al., 2019; Browne et al., 2016; Korn & Shaffer, 1999; Livingstone & Rintoul, 2020; van Schalkwyk, Cassidy, McKee, & Petticrew, 2019; Wardle, Reith, Langham, & Rogers, 2019). Safer gambling messaging is one potential input to a public health approach toward this goal (Gambling Commission, 2019), similar to the messages used across related public health issues of tobacco and alcohol. Electronic gambling machines (EGMs) are one gambling product that contributes disproportionately to the public’s gambling losses in many jurisdictions (The Economist, 2017), and which has been the object of a range of research into safer gambling messaging (Ginley et al., 2017). In contrast, race and sports betting are growing contributors to gambling losses in multiple jurisdictions (Altruda, 2022; Queensland Government, 2019), likely due to their migration to frequently-advertised online and mobile platforms (Hing et al., 2015, 2016; Newall, Russell, & Hing, 2021). However, race and sports betting has been associated with far less research on safer gambling messaging. Consequently, evidence needs to be sourced from other gambling activities and related public health literature on areas such as problem drinking. The purpose of this review is to consider what can be learned from the existing gambling and public health literature that might inform the design of improved product-safety messages for race and sports betting.

Despite sports and race wagering being the main form of online gambling and the greatest source of online gambling harm in Australia (Hing et al., 2022), there is no consistent national approach to safer gambling messages, aside from the common imploration, mandated for use by gambling providers, for consumers to “gamble responsibly”. A recent eye-tracking study suggests that these messages either (a) are repeated identically so often or (b) take up such a small part of physical space in Australian gambling advertisements, that regular sports bettors rarely absorb them (Lole et al., 2019). A very reasonable proposal is therefore that these “gamble responsibly” messages should be made larger in size, similar to that required of regulated Australian tobacco warnings over time (Borland & Hill, 1997).

However, some researchers doubt the effectiveness of “gamble responsibly” because of the message’s implications. A plea to gamble responsibly implies that some fraction of gamblers gamble irresponsibly (Reith, 2008), and that gambling-related harm is therefore a consequence of a lack of individual responsibility. Such messages that implicate personal failings may generate gambling-related stigma (Hing et al., 2016; Miller & Thomas, 2017), which could have unintended consequences in relation to shame and stigma, and reduce the likelihood of harm-reducing behaviours such as help-seeking. This effect of message backfiring has been observed experimentally in the alcohol literature with the corresponding “drink responsibly” message (Moss et al., 2015).

Although we know of no research on whether “gamble responsibly” has any positive or negative effects on help-seeking or other gambling behaviours, there is one robust psychological reason beyond mere inattention, to consider a range of safer gambling messages. The frequent repetition of identical messages has been shown by social psychologists to lead to negative psychological reactance, whereby the message backfires and has an opposite effect to the one that was intended (Cacioppo & Petty, 1979). This negative psychological reactance may well have, in part, led to the backfire effects observed with the “drink responsibly” message, given the similar ubiquity of that message in the alcohol domain.

Individual differences that influence the effectiveness of messages are another reason to consider multiple potential safer gambling messages. In fact, many studies have shown that generic slogans, particularly delivered on signage within venues, are largely ignored by gamblers (Monaghan & Blaszczynski, 2010; Hing, 2003; Schrans, Grace & Schellinck, 2003). Some gamblers may be more impacted by specific safer gambling tips or strategies, while other gamblers may be more impacted by a message with emotional content. Such stable individual differences in specific warning effectiveness have been observed in the graphic health warning tobacco literature (Romer et al., 2013). Other research suggests that safer gambling messages can also backfire, if delivered to the wrong part of the population (Armstrong et al., 2018; Mizerski et al., 2012). It is therefore important to consider many different types of safer gambling message, and to explore how each message of these messages is received amongst different groups of gamblers, given the wide range of gamblers’ motives (Stewart & Zack, 2008) and the different stages of change of those wanting to manage their gambling (Prochaska & DiClemente, 1986).

Safer gambling messages are in use in other jurisdictions beyond Australia but have drawn similar criticisms to that of “gamble responsibly”, for example, “keep the fun in the game” in Ontario, Canada (Newall et al., 2022), and “so that gambling remains a game” in Switzerland (Mouneyrac et al., 2017). These messages, that implicitly highlight that gambling is a “game”, are similar to the gambling industry’s preferred term of “gaming”, in replacement for “gambling” (Reith, 2008). A safer gambling message which highlights that gambling is a game, and therefore creates connotations of innocuous play, has arguably poor face validity. The Canadian message’s additional use of the word “fun” is also not an isolated example of the use of this word in safer gambling messages. “When the fun stops, stop” was the most common UK safer gambling message from its inception in 2015 to 2021. The only set of independent evaluations of that message, using contemporaneous gambling behaviour as a dependent variable, found results consistent with a small backfire effect, whereby participants gambled slightly more often in the presence of the main version of that message, where the word “fun” is written in larger font than the rest of the text (Newall et al., 2022). This suggests that there are few credible safer gambling messages currently in use internationally that are based on either sound face validity or demonstrably positive behavioral effects.

The remainder of this review will proceed by first considering related literature from other public health domains, before describing five potential message themes based on previous gambling research: teaching safer gambling practices, correcting gambling misperceptions, boosting conscious decision making, norm-based messages, and emotional messages.

## Comparisons with Other Public Health Domains

The most effective health messages on consumer behaviour have perhaps been developed in tobacco: prominent health warnings on tobacco packages can help consumers to quit smoking (Hammond, 2011). In tobacco, warning prominence has been shown to be relevant, with larger warnings, and in particular larger graphic warnings of smoking’s consequences, being the most effective (Hammond, 2011; Noar et al., 2016). However, it has also been suggested that daily smokers, who consume the most tobacco, might pay the least attention to tobacco warnings (Maynard et al., 2013; Munafò, Roberts, Bauld, & Leonards, 2011), suggesting that product warnings are only one part of a public health approach to reduce harm from tobacco.

Alcohol containers also have to contain health warnings in many jurisdictions, such as warnings about drinking while pregnant or driving (Hilton, 1993). However, these warnings have been criticised for their lack of prominence (Hilton, 1993; Kersbergen & Field, 2017), the extent to which the alcohol industry has failed to promote effective warnings (Petticrew et al., 2016; Stockwell, Solomon, O’Brien, Vallance, & Hobin, 2020), and current warnings’ lack on influence on consumers (Hilton, 1993). Cancer health warnings have also been investigated experimentally in alcohol (Pettigrew et al., 2014). Of the various text-based cancer health warnings tested, “Alcohol increases your risk of bowel cancer” was found to be the most effective in one study, with participants rating it as the most believable, convincing, and personally relevant message (Pettigrew et al., 2016). Graphic warnings around alcohol’s health risks have also been trialled to mimic the graphic warnings in current use in tobacco. For example, one experiment tested the addition of a picture of a diseased liver to a tobacco-style warning saying “Alcohol causes fatal liver cancer” (Wigg & Stafford, 2016). The addition of this diseased liver picture was associated with greater levels of fear arousal and an intention to reduce and quit consuming alcohol, compared to a text-only warning, replicating the results from tobacco (Hammond, 2011).

Although alcohol messaging is generally considered helpful, researchers have illustrated ways that alcohol messaging might backfire and provide a result that is opposite from what is intended (Moss et al., 2015). For example, the addition of the US alcohol warning to mock advertisements in an experiment led to greater perceptions of benefits of drinking (Snyder & Blood, 1992). As another example, “licensing effects” that encourage higher levels of consumption can occur when a given container is described as “low alcohol.” These labels might encourage consumers to consume more of the product that they otherwise would, potentially leading to an overall increase in pure ethanol consumed (Shemilt et al., 2017). Standard drink information, which informs about the alcohol content of a given container meanwhile, could help teenager binge-drinkers to find the most cost-effective drinks to get drunk, and therefore help them to consume as much alcohol as they possibly can (Wells et al., 2009).

Evidence on warnings about gambling-related harm is noticeably absent compared to evidence on alcohol and tobacco warning labels. This could potentially be due to the lack of a strong evidence base regarding the proximal causal mechanisms for excessive consumption and associated harm, relative to other conditions. Tobacco’s health effects have been documented over decades of epidemiological research, and alcohol is also in the top five risk factors for disease and disability worldwide (Hobin et al., 2017). In addition, tobacco’s health effects are viscerally evident, which simplifies the production of impactful graphic tobacco warnings, and a similar argument can be made for graphic alcohol warnings (Wigg & Stafford, 2016). In short, the substance-based behavioural disorders present a simpler aetiological challenge for researchers, which provides the basis for more clear-cut warnings to consumers regarding what constitutes harmful use. By comparison, rigorous empirical evidence around gambling-related harm has only begun to emerge in the last few years (Browne et al., 2016; Markham, Young, & Doran, 2016; Muggleton et al., 2021). Whilst risk to human physiology from alcohol and tobacco is relatively constant across individuals, the intensity threshold for harmful gambling is highly dependent on a person’s available time and financial resources. Further, the types of harms associated with gambling are not as graphic as those from tobacco or alcohol, meaning that there are fewer opportunities for high impact images. Pathways from gambling to harm are also much more indirect than the effects that tobacco and alcohol have on the body. It appears that financial losses are the core driver of gambling-related harm, but health and wellbeing are ultimately affected across multiple life domains (Langham et al., 2016). This includes via effects on non-gamblers and significant others (Goodwin et al., 2017; Tulloch et al., 2021, 2022), for example, through increases in domestic violence (Hing et al., 2021; Markham, Doran, & Young, 2016). The multiplicity of types of gambling-related harm may mean that the average disordered gambler has only experienced a fraction of gambling’s possible harmful effects (Browne & Rockloff, 2018; Delfabbro & King, 2019). This makes identifying and communicating the ‘signs’ of harmful gambling more challenging.

The indirect nature of gambling-related harm may make it harder to construct safer gambling messages analogous to health warnings in tobacco and alcohol that gamblers find believable and personally relevant. One proposed textual message warning of the potential effects of gambling-related harm is, ‘Gambling is associated with significant harms including increased risks of physical and mental health problems, separation, divorce, financial difficulties and bankruptcy, intimate partner violence and fraud’ (Livingstone et al., 2019, p.10). However, such a warning may be ignored by the many gamblers who have not experienced such effects (Browne & Rockloff, 2018), and may be an insufficiently strong intervention for those who have (Delfabbro & King, 2020).

Tobacco and alcohol messages may also influence behaviour via effects mediated by emotion. Graphic alcohol warnings tested experimentally elicit increased levels of fear (Wigg & Stafford, 2016). A recent meta-analysis from the tobacco literature suggests that effective graphic warnings use the channels of fear and negative emotions (Noar et al., 2020), a view that an even more recent study supports (Sillero-Rejon et al., 2020). It has also been suggested in the wider behaviour change literature that these negative emotion and fear appeals have the strongest effects when they also maintain perceptions of self-efficacy, i.e., that people have the power to change (Witte & Allen, 2000). In contrast, messages can also attempt to leverage the power of positive emotions, such as hope or humour. In tobacco, anti-smoking campaign adverts using humour were rated as being less effective by smokers, non-smokers, and smokers planning to quit than adverts eliciting sadness and fear (Biener et al., 2000). However, a study on alcohol messaging suggests that campaigns targeting positive emotions such as happiness and love have a better chance of supporting intentions to reduce alcohol consumption than negative emotion campaigns (Previte, Russell‐Bennett, & Parkinson, 2015).

These differing effects of emotional messages in tobacco and alcohol complicate the potential use of emotional safer gambling messages. There are plenty of possible negative effects of gambling (Langham et al., 2016; Muggleton et al., 2021), and a message harnessing these could be effective (Mutti-Packer et al., 2022). However, gambling is associated with low levels of help-seeking (Suurvali et al., 2008), and increased perceived social stigma (Hing & Russell, 2017; Horch & Hodgins, 2008), and feelings of shame (Yi & Kanetkar, 2011). It could therefore be that negative-emotion gambling messages will lead to avoidance and fail to encourage positive behavioural impacts. This view would suggest that positive-emotion gambling messages may be more effective.

## Potential Safer Gambling Message Themes

### Teaching Safer Gambling Practices

One logical input for safer gambling messages is to provide information on the behaviours correlated most strongly with non-harmful gambling. Some previous research has investigated the safer gambling practices highlighted in previous gambling research and in gambling information online (Hing et al., 2019). In all, 51 safer gambling practices were found. These 51 safer gambling practices were then used to predict actual levels of gambling-related harm in a sample of 577 gamblers who were susceptible to harm. This procedure led to nine safer gambling practices that best predicted low levels of gambling-related harm, including ‘I have a dedicated budget to spend on gambling’, ‘If I’m feeling depressed or upset, I don’t gamble’, and ‘When I gamble, I always set aside a fixed amount to spend.’

These candidate items therefore are potentially valid as candidate safer gambling messages. However, their actual effectiveness as a part of safer gambling messages is not guaranteed. The procedure for selecting candidate safer gambling messages may have missed some effective safer gambling practices. Any safer gambling practices endorsed by gamblers experiencing low levels of harm may simply be correlates of harm, and if they have no causal influence in reducing gambling-related harm, may therefore not be effective strategies to teach to gamblers. Additionally, the effectiveness of any safer gambling practice depends not only on the gambler but also on their decision environment. For example, setting aside a dedicated budget to spend on gambling may only work in a setting where a gambler has access to a binding precommitment option; if other gambling funds can be readily accessed, for example in online betting, then it may be hard to put this action into practice. Avoiding gambling when depressed or upset may be possible only in environments with low levels of gambling marketing; exposure to marketing cues may prompt some gamblers to bet even when they intend not to.

A more recent study addressed some of these limitations through a randomised controlled trial (Hing et al., 2022). The study demonstrated reductions in EGM spend and gambling harm when safer gambling practices were recommended to frequent EGM gamblers who subsequently implemented them. These practices were: setting aside a fixed amount to spend; taking regular breaks; keeping leisure time busy with other activities; not gambling due to boredom; and keeping a household budget. However, the authors noted that these strategies are likely to be of most use to low or moderate risk gamblers. Individuals with a severe gambling disorder are likely to need additional and stronger strategies, including professional help, to implement protective measures and to resolve their gambling problem.

Moore, Thomas, Kyrios and Bates (2012) similarly examined 27 self-management techniques that they found were more often used with people experiencing gambling problems. Exploratory factor analysis discovered 5 factors, including Cognitive Approaches (e.g., think about consequences), Direct Action (e.g., getting help), Social Experience (e.g., avoid gambling alone), Avoidance (e.g., avoiding casinos), and Limit Setting. To the extent that these practices are logically or verifiably related to a reduction in gambling-related harm, they are useful source materials for messaging.

### Correcting Gambling Misperceptions

Corrective thought messaging emerged from the cognitive perspective on gambling, the observation that disordered gamblers tend to have many incorrect beliefs about luck and random chance (Raylu & Oei, 2004; Walker, 1992). The cognitive perspective has successfully informed effective clinical psychological treatments for disordered gamblers (Ladouceur et al., 2001; Petry et al., 2006). The cognitive perspective has also informed the design of corrective thought messages, which aim to correct gamblers’ incorrect thoughts during gambling to hopefully modify their behaviour. Corrective thought messages have mainly been applied thus-far to EGMs, and include content such as, “No strategy will enable you to win more often” (Cloutier et al., 2006). As with the tobacco literature, research on corrective thought messages highlights the importance of having salient messages, which in the case of EGMs means messages in the centre of the display, occurring during a break in play, which require active engagement to restart play (Ginley et al., 2017).

One issue with corrective thought messaging is that there is a multiplicity of incorrect thoughts that any gambler can have. A successful corrective thought message must therefore target an incorrect thought that the gambler actually has, be taken on board by the gambler, and then lead to a subsequent behavioural change. This issue can be demonstrated via the number of items in gambling misperception self-report scales, which one review demonstrates can have between 10 and 56 individually scored items (Leonard et al., 2015). However, some frequently-occurring gambling misperceptions are that many gamblers are motivated “to win money” (Gambling Commission, 2018), and that systems or strategies can help a gambler to achieve this aim (Raylu & Oei, 2004; Walker, 1992).

A further issue with attempting to correct such potential misperceptions is that gambling forms differ with respect to the balance between skill and luck. Sports and race wagering are potentially some of the highest potential skilled forms of gambling, with one estimate suggesting there is more skill involved in being a successful sports bettor than in being a successful mutual fund manager (Getty et al., 2018). It is possible that a message that downplays the potential for sports bettors to make money in the long run could be received negatively by a fraction of the audience. This means that a number of the corrective thought messages applied to EGMs may be inappropriate for these race and sports betting (Cloutier et al., 2006). More broadly, it has been highlighted that the research base on gambling misperceptions is largely built on luck-based gambling forms such as EGMs (Russell et al., 2019). This means that any translation of the gambling misperception literature to sports and race wagering should be performed cautiously.

Additionally, researchers have highlighted that although corrective thought messages are effective at altering gamblers’ knowledge, they are less effective at improving gamblers’ behaviours, even within the EGM domain (Monaghan & Blaszczynski, 2009). A recent large scale prospective study analysed the time course of gambling misperceptions, gambling engagement, and disordered gambling (Leonard et al., 2021). Importantly, that study found that although gambling misperceptions co-occur with disordered gambling, they do not appear to be the main driver of disordered gambling. That study concluded that correcting gambling misperceptions should not be the sole aim of gambling treatment. It is therefore reasonable to consider that they should also not be the main content type of safer gambling messages.

### Boosting Conscious Decision Making

Given the potential limited effectiveness of correcting gamblers’ misperceptions (Monaghan & Blaszczynski, 2009), other related approaches should also be considered. One recent proposal is to try to boost gamblers’ levels of conscious decision making by encouraging analytical thinking (Armstrong et al., 2020). This proposal was tested in an experimental 4-week intervention (*N* = 94), investigating the effect of providing gamblers with feedback on responses to a total of 50 gambling fallacies questions. Each question had a normatively correct response based on statistical logic. The intervention involved providing treatment-group participants with the correct response and the statistical logic underlying that response.

For example:

“Tracey likes to bet on the winner of the local football match. Her last three picks have all won the games. In the next game, the 2 teams are equally matched. All else being equal, what are the odds that her pick for the next match will also win the game?

- 50%.
- More than 50%.
- Less than 50%.

Feedback: While Tracey may be knowledgeable on football, in this instance the teams are evenly matched and therefore, it is irrelevant whether or not Tracey has some football expertise. Each gamble is independent from the last so for her next gamble, the odds of picking a winner out of two otherwise equal opponents is still 50/50.” (Armstrong et al., 2020, pp. 781–782).

By comparison, participants in the control condition simply answered gambling trivia questions without receiving any feedback at all. Analysis of the intervention suggested that treatment-group participants saw a significantly greater improvement in scores on the predictive control subscale of the Gambling Related Cognition Scale (Raylu & Oei, 2004) than control-group participants. Plausibly, a safer gambling message could be constructed based on this example item that informs gamblers that being on a “lucky streak” does not improve the odds of winning on their next bet. However, although this study showed impacts on beliefs, it was not able to provide compelling evidence that this belief change manifested into behavioural change (Armstrong et al., 2020).

### Norm-Based Messages

Social psychology has shown repeatedly that other people’s behaviour can be a strong influence on how we act. For example, one classic example of how this motivation can backfire was a finding that around 8% of visitors to a national park stole petrified wood chips from a national forest when given a sign that read, ‘Many past visitors have removed the petrified wood from the park, changing the state of the petrified forest’, compared to under 2% when the sign read, ‘Please don’t remove the petrified wood chips’ (Cialdini et al., 2006). This is because the first sign normalised the undesired behaviour; the effect of any individual’s actions was therefore diluted by the perception of the crowd’s behaviour.

Some research has explored related social messages in field studies run in collaboration with online wagering operators. In one study, gamblers were provided feedback that they were spending more time than others gambling online, e.g., “You have spent more than X hours playing over the last Y days. Most customers played for no more than Z hours in that time” (Behavioural Insights Team, 2018, p.67). This could be seen as a norm-based intervention, which Behavioural Insights Team field studies have found effective in changing other aspects of consumer behaviour (Behavioural Insights Team, 2012). This message was added to an email sent to actual betting account owners from their online operator. The norm-based messages on time spent gambling were not effective in increasing participants’ uptake of safer gambling tools, however (Behavioural Insights Team, 2018). Another study using data collected with online operators investigated the effects of a social message highlighting the popularity of deposit limiting tools, “Most people who use deposit limits find this helps them manage their spending”. Although this message led to more wagering customers setting deposit limits, it was no more effective than a control message merely informing them about the presence of a deposit limit setting tool (Heirene & Gainsbury, 2021). Another study, conducted independent of the gambling industry, tested a message to set wagering limits (Hing et al., 2021). The message was developed through a discrete choice experiment that identified optimal message features in relation to type of limit, terminology and purpose, information to help set limits, message personalisation, message framing and message targeting. A 4-week RCT found that receiving the optimal message had no significantly better effect on participants’ attitudes towards intention to set, or actually setting of a deposit limit; including for the weekly vs. fortnightly messages and personalised vs. non-personalised messages.

However, another trial initiated via an online operator found better results. This intervention was a message delivered to gamblers who played 1,000 spins on an online slots product. Control-group participants were simply told that they had played 1,000 spins, whereas treatment-group participants were further told that:

“Only a few people play more than 1,000 slot games. The chance of winning does not increase with the duration of the session. Taking a break often helps, and you can choose the duration of the break.” (Auer & Griffiths, 2015, p.3).

Results showed that this message doubled the proportion of gamblers quitting from 0.7 to 1.4%, a significant increase over the sample size of 1.6 million sessions. The norm-based element of this message is the first sentence, that only a few people play more than 1,000 slot games. This message does however contain other information, such as information to potentially correct a gambling misperception (“The chance of winning does not increase with the duration of the session.”), and information on a safer gambling practice (“Taking a break often helps, and you can choose the duration of the break”). Furthermore, although the result was statistically significant, the absolute proportion of gamblers quitting was still small (1.4%).

Results of one study showed that personalised feedback on how an at-risk gambler’s expenditure compares to their peers’ expenditure led to a significant reduction in gambling frequency (Larimer et al., 2012). A meta-analysis on the effect of providing personalised feedback on how a gambler’s behaviour differs from that of their peers’ behaviour also showed significant effects (Peter et al., 2019). However, these interventions were heterogenous and could each contain multiple elements, introducing a confounding issue. Of relevance to safer gambling messages, the element of personalisation is necessarily absent from any population-based messaging approach. Therefore, a norm-based safer gambling message can only provide information on population averages and must depend on an individual to know their own situation well enough to make an accurate comparison with the average.

One solution to this inability to provide personalised comparisons is to provide particularly extreme population figures. This extreme figurecan therefore be inferred to apply to most gamblers receiving the message. For example, the Chief Executive of a large UK-based gambling operator gave the following information to a House of Lords Select Committee: “99% of the customers who play on our sites will lose, so you’re probably losing more if you play more.” (Alexander, 2020). Although more than 1% of gamblers are likely to think that they are in the 1% of gamblers that do not lose, this extreme population figure should still appear relevant to many gamblers. Similarly, population figures on gambling expenditure could be given, which when provided on an aggregate level can amount to large amounts, and which will appear relevant to most gamblers. Current figures put the Australian public’s gambling losses at $24.8 billion a year (Wallace, 2021).

Another approach may be to use norm-based information that may be surprising to many recipients. For example, it has been found that a problem gambler harms on average six of their close family and friends (Goodwin et al., 2017). Similarly, population-based prevalence surveys typically find that the problem gambling base rate is around 1% (Collins et al., 2020), and so many gamblers may think that is an appropriate base rate for gambling-related harm, as well. However, some recent estimates suggest this proportion might be much higher --- potentially as high as one in five gamblers (Rockloff et al., 2020) or around 10% of the overall population.

A benefit to using either extreme or surprising norm-based information is that these may help reduce the likelihood of these messages backfiring. Recall how a study mentioned above found that a message that implied that an undesired behaviour was nonetheless common led to increases in the behaviour (Cialdini et al., 2006). It is possible with norm-based messages that a gambler may, for example, see that their expenditure is below population averages, and therefore feel comfortable gambling more. However, the messages proposed over the previous two paragraphs should lessen the possibility of this backfire effect occurring.

### Emotional Messages

Previous research has investigated the effects of safer gambling messages leveraging negative emotions. In one study (Munoz et al., 2010), the highly-threatening message, ‘Excessive gambling may drive you to intense distress and suicidal thoughts’ was compared to a control condition message of, ‘gambling should remain a game’, which mimics some of the safer gambling messages in current use (Mouneyrac et al., 2017). This study found that the message did induce fear and was able to make gamblers think more deeply about their behaviour (Munoz et al., 2010). A follow-up study explored the effects of adding a graphic warning label to the fear-inducing text also intending to induce fear (Munoz et al., 2013). The graphic used involved a cartoon of a gambler being eaten by an EGM --- which is quite different to the graphic warnings used in tobacco (Noar et al., 2020) and which have been trialled in alcohol use (Wigg & Stafford, 2016). Results of this study supported the previous gambling study, suggesting that the addition of a graphic to fear-inducing text helped to further increase feelings of fear and the extent to which gamblers thought about their behaviour (Munoz et al., 2013). However, one qualitative study revealed that many gamblers thought that excessively fear-inducing stimuli may be experienced as stigma-inducing and may be ineffective due to avoidance (De Vos et al., 2017). This is a plausible alternative explanation, at least for some groups, which the earlier studies’ lack of behavioural measures of help-seeking or reduction in gambling expenditure cannot rule out (Munoz et al., 2010, 2013).

By contrast, there does not appear to be any empirical research on the role of safer gambling messages which induce positive emotions. One recent opinion piece states that gambling researchers should attempt to address this, given that positive emotional messages have had some success in other public health domains (Harris et al., 2018). In particular, one suggested message was as follows, ‘save the rest of your money for that family trip next month’ (Harris et al., 2018, p.271).

## Conclusion

This review has considered the relevant lessons that can be applied for safer gambling messages in race and sports wagering from alcohol, tobacco, and previous gambling research. Lessons from previous research included avoiding very simple messages which are repeated often, and the importance of testing messages in order to show that they do not lead to backfire effects either across the whole population or within certain subgroups. Finally, the review concluded with five potential message themes based on previous gambling research as worth consideration with respect to designing improved safer gambling messages for race and sports betting: teaching safer gambling practices, correcting gambling misperceptions, boosting conscious decision making, norm-based messages, and emotional messages.

## Data Availability

Data sharing not applicable to this article as no datasets were generated or analysed during the current study.
